# Malaria morbidity and mortality following introduction of a universal policy of artemisinin-based treatment for malaria in Papua, Indonesia: A longitudinal surveillance study

**DOI:** 10.1371/journal.pmed.1002815

**Published:** 2019-05-29

**Authors:** Enny Kenangalem, Jeanne Rini Poespoprodjo, Nicholas M. Douglas, Faustina Helena Burdam, Ketut Gdeumana, Ferry Chalfein, Prayoga, Franciscus Thio, Angela Devine, Jutta Marfurt, Govert Waramori, Shunmay Yeung, Rintis Noviyanti, Pasi Penttinen, Michael J. Bangs, Paulus Sugiarto, Julie A. Simpson, Yati Soenarto, Nicholas M. Anstey, Ric N. Price

**Affiliations:** 1 Timika Malaria Research Program, Papuan Health and Community Development Foundation, Timika, Papua, Indonesia; 2 Mimika District Health Authority, Timika, Papua, Indonesia; 3 Pediatric Research Office, Department of Child Health, Faculty of Medicine, Public Health and Nursing, Universitas Gadjah Mada, Yogyakarta, Indonesia; 4 Rumah Sakit Umum Daerah Kabupaten Mimika, Timika, Papua, Indonesia; 5 Global and Tropical Health Division, Menzies School of Health Research, Charles Darwin University, Darwin, Australia; 6 Public Health & Malaria Control Department, PT Freeport Indonesia/International SOS, Kuala Kencana, Papua, Indonesia; 7 Centre for Epidemiology and Biostatistics, Melbourne School of Population and Global Health, University of Melbourne, Australia; 8 Faculty of Infectious and Tropical Diseases, London School of Hygiene & Tropical Medicine, London, United Kingdom; 9 Eijkman Institute for Molecular Biology, Jakarta, Indonesia; 10 European Centre for Disease Prevention and Control, Solna, Sweden; 11 Department of Entomology, Faculty of Agriculture, Kasetsart University, Bangkok, Thailand; 12 Rumah Sakit Mitra Masyarakat, Timika, Papua, Indonesia; 13 Centre for Tropical Medicine and Global Health, Nuffield Department of Clinical Medicine, University of Oxford, Oxford, United Kingdom; 14 Mahidol-Oxford Tropical Medicine Research Unit (MORU), Faculty of Tropical Medicine, Mahidol University, Bangkok, Thailand; Mahidol-Oxford Tropical Medicine Research Unit, THAILAND

## Abstract

**Background:**

Malaria control activities can have a disproportionately greater impact on *Plasmodium falciparum* than on *P*. *vivax* in areas where both species are coendemic. We investigated temporal trends in malaria-related morbidity and mortality in Papua, Indonesia, before and after introduction of a universal, artemisinin-based antimalarial treatment strategy for all *Plasmodium* species.

**Methods and findings:**

A prospective, district-wide malariometric surveillance system was established in April 2004 to record all cases of malaria at community clinics and the regional hospital and maintained until December 2013. In March 2006, antimalarial treatment policy was changed to artemisinin combination therapy for uncomplicated malaria and intravenous artesunate for severe malaria due to any *Plasmodium* species. Over the study period, a total of 418,238 patients presented to the surveillance facilities with malaria. The proportion of patients with malaria requiring admission to hospital fell from 26.9% (7,745/28,789) in the pre–policy change period (April 2004 to March 2006) to 14.0% (4,786/34,117) in the late transition period (April 2008 to December 2009), a difference of −12.9% (95% confidence interval [CI] −13.5% to −12.2%). There was a significant fall in the mortality of patients presenting to the hospital with *P*. *falciparum* malaria (0.53% [100/18,965] versus 0.32% [57/17,691]; difference = −0.21% [95% CI −0.34 to −0.07]) but not in patients with *P*. *vivax* malaria (0.28% [21/7,545] versus 0.23% [28/12,397]; difference = −0.05% [95% CI −0.20 to 0.09]). Between the same periods, the overall proportion of malaria due to *P*. *vivax* rose from 44.1% (30,444/69,098) to 53.3% (29,934/56,125) in the community clinics and from 32.4% (9,325/28,789) to 44.1% (15,035/34,117) at the hospital. After controlling for population growth and changes in treatment-seeking behaviour, the incidence of *P*. *falciparum* malaria fell from 511 to 249 per 1,000 person-years (py) (incidence rate ratio [IRR] = 0.49 [95% CI 0.48–0.49]), whereas the incidence of *P*. *vivax* malaria fell from 331 to 239 per 1,000 py (IRR = 0.72 [95% CI 0.71–0.73]). The main limitations of our study were possible confounding from changes in healthcare provision, a growing population, and significant shifts in treatment-seeking behaviour following implementation of a new antimalarial policy.

**Conclusions:**

In this area with high levels of antimalarial drug resistance, adoption of a universal policy of efficacious artemisinin-based therapy for malaria infections due to any *Plasmodium* species was associated with a significant reduction in total malaria-attributable morbidity and mortality. The burden of *P*. *falciparum* malaria was reduced to a greater extent than that of *P*. *vivax* malaria. In coendemic regions, the timely elimination of malaria will require that safe and effective radical cure of both the blood and liver stages of the parasite is widely available for all patients at risk of malaria.

## Abstract 2

Ric Price and colleagues report on changes in Plasmodium falciparum and vivax malaria following a universal malaria treatment programme in Papua, Indonesia.

## Author summary

**Why was this study done?:**

- Multidrug-resistant malaria results in recurrent parasitaemia, a cumulative risk of anaemia, and progression to severe and fatal disease.
- Whilst artemisinin combination therapies (ACTs) and intravenous (IV) artesunate can reduce morbidity and mortality associated with *P*. *falciparum* malaria, they have no activity on the hypnozoite stages of *P*. *vivax*, which can relapse weeks to months following an initial infection and sustain ongoing transmission of the parasite.
- In Papua, Indonesia, antimalarial resistance has emerged in both *P*. *falciparum* and *P*. *vivax*. We used a prospective malariometric surveillance network to investigate the differential impact of a universal policy of ACTs for uncomplicated malaria and IV artesunate for severe malaria on the morbidity and mortality attributable to *P*. *falciparum* and *P*. *vivax*.

**What did the researchers do and find?:**

- After controlling for population growth and changes in treatment-seeking behaviour, the incidence of malaria after policy change fell by about 60% for *P*. *falciparum* and 40% for *P*. *vivax*.
- There was a 50% fall in the proportion of patients with malaria requiring admission to hospital and a 30% fall in malaria-related mortality. Bed occupancy due to admission with malaria fell by 25%.
- Whilst there was a small decrease in the absolute incidence of *P*. *vivax* infections over the 9-year study period, the proportion of malaria cases and malaria-attributable deaths to *P*. *vivax* increased with time.

**What do these findings mean?:**

- The results highlight the importance of highly effective blood schizontocidal antimalarial drugs in reducing the overall burden of drug-resistant malaria.
- The rising proportion of malaria due to *P*. *vivax* emphasizes the need for safe and effective drug regimens that clear both the blood and liver stages of *P*. *vivax* in malaria elimination efforts in coendemic regions.

## Introduction

Prompt and effective treatment of malaria reduces morbidity and limits onward transmission of the *Plasmodium* parasite [1,2]. Large-scale use of highly efficacious antimalarial treatment regimens has contributed to significant reductions in *P*. *falciparum* malaria in many malaria-endemic regions [3,4]. *P*. *vivax* is more difficult to cure than *P*. *falciparum* because it forms dormant liver stages (hypnozoites) that are intrinsically resistant to standard schizontocidal drugs. Unless patients are treated with an effective drug regimen that clears both the blood and liver stage of the parasite, these hypnozoites can reactivate periodically, causing recurrent blood-stage infections (relapses) and ongoing transmission [5]. Malaria treatment campaigns that do not include radically curative primaquine regimens for patients infected with *P*. *vivax* may have only a modest effect on the number of cases of *P*. *vivax* malaria and thus are likely to be associated with an increase in the proportion of malaria due to this parasite compared to *P*. *falciparum* [6–8]. When primaquine radical cure is included in national guidelines, it is usually prescribed without prior testing for glucose-6-phosphate dehydrogenase (G6PD) deficiency. To mitigate the risks of drug-induced haemolysis, many countries recommend a 15-mg daily dose administered over 14 days despite evidence showing that 30 mg daily is more effective [9]. When supervised, a 14-day regimen of primaquine can reduce the risk of *P*. *vivax* relapse by more than 85% [10,11]; however, in most endemic settings, daily supervision of such a prolonged treatment regimen is not practical [12], and this can result in a significant reduction in primaquine adherence and effectiveness [13–15]. In a large-scale observational study of patients with vivax malaria in Papua, Indonesia, the effectiveness of unsupervised primaquine was estimated to be only 10% [16].

Malaria endemicity in Papua, Indonesia, varies from hypo- to hyperendemic for *P*. *falciparum* and *P*. *vivax* [17]. In the early 2000s, clinical trials and ex vivo drug-susceptibility testing demonstrated high-grade resistance to chloroquine and sulphadoxine + pyrimethamine in endemic *P*. *falciparum* strains and chloroquine resistance in *P*. *vivax* strains [18–20]. Frequent, recurrent parasitaemia is more likely in the setting of high-grade drug resistance and is associated with a cumulative risk of chronic anaemia, severe malaria, and mortality [21,22]. Therefore, in March 2006, Indonesian national antimalarial treatment guidelines were changed to an artemisinin combination therapy (ACT) (dihydroartemisinin plus piperaquine [DP]) for uncomplicated malaria due to any *Plasmodium* species and intravenous (IV) artesunate for severe malaria. At the same time, policy for the use of primaquine in patients with *P*. *vivax* infections was changed from a total dose of 3.5 mg/kg over 14 days to a higher dose of 7 mg/kg over 14 days. Information regarding the treatment changes was distributed widely via health professionals and community leaders. In Mimika District, located in southern Papua Province, hospital and community surveillance systems were put into place prior to the policy change to allow an assessment of the subsequent changes in malaria-attributable morbidity and mortality due to either *P*. *falciparum* or *P*. *vivax*. Previous analyses from the same location have quantified the burden of malaria in the hospital, the epidemiology of malaria in the community, and local treatment-seeking behaviour [17,22,23]. The current analysis used routinely collected surveillance data collected over a 9-year period (2004 to 2013) to investigate the temporal trends in malaria morbidity and mortality before and after the change in antimalarial treatment policy and the relative impact of this intervention on the burden of *P*. *falciparum* compared with *P*. *vivax*.

## Methods

### Study site

**Fig 1:**
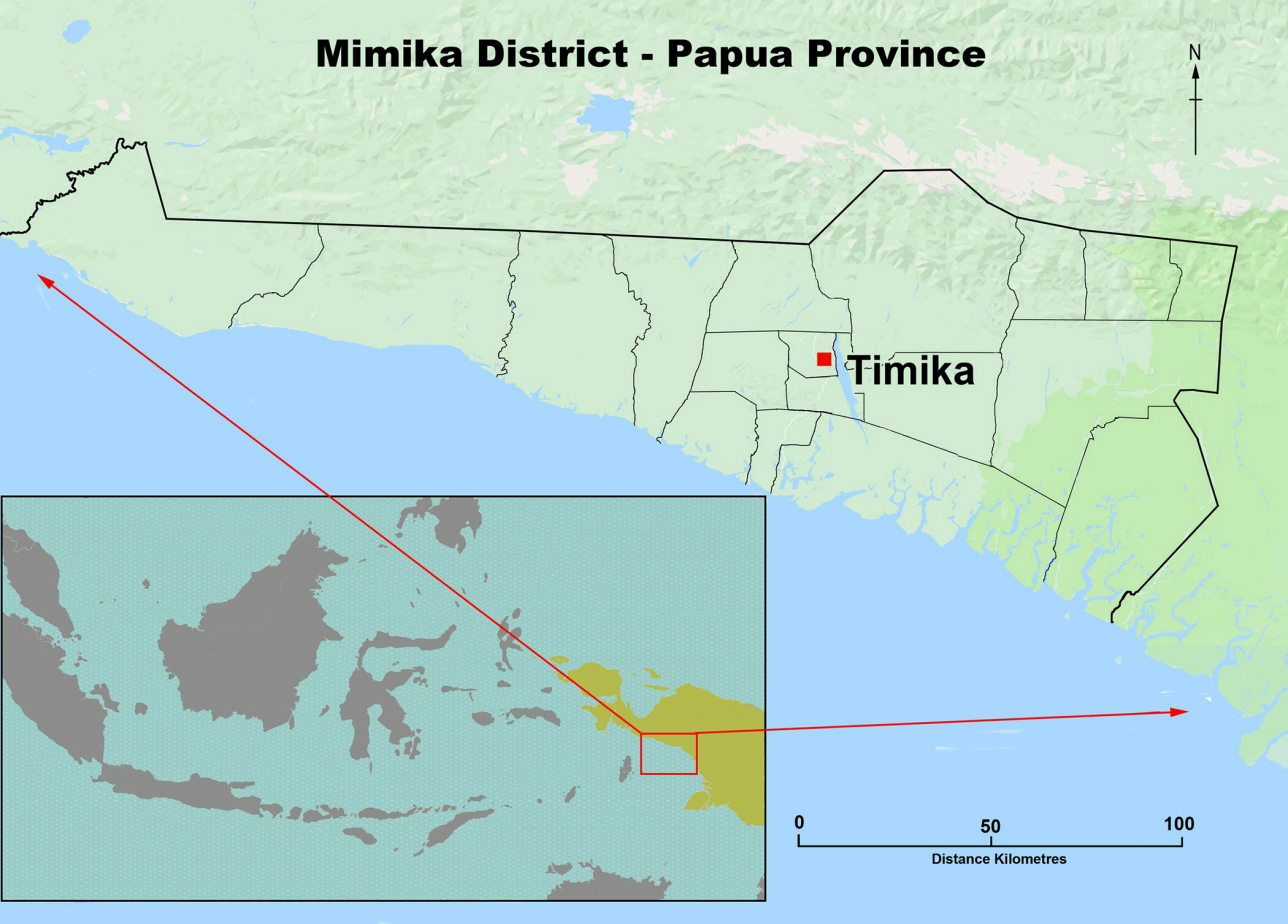
Map of Mimika district in Papua Province, eastern Indonesia. Map adapted from WorldOfMaps (https://www.worldofmaps.com).

The geography, climate, and demographics of Mimika District and its capital, Timika, have been described previously [17,22]. Briefly, Mimika District lies in south central Papua, eastern Indonesia, and covers an area of 21,522 km^2^; it has 12 subdistricts and 85 villages (Fig 1). The region has fragmented forest ranging from extensive coastal lowlands to high mountainous environments. Timika has a growing population of native Papuans and Indonesian migrants, estimated to be 120,457 in 2004 and increasing to 196,401 in 2013 [24].

Malaria transmission is perennial with minimal seasonal variation and is normally restricted to the lowland areas below 1,600-m elevation, where most of the population now resides. There are three primary mosquito vectors: *Anopheles koliensis*, two members of the *A*. *farauti* complex, and *A*. *punctulatus*; all of these vectors are both exo- and endophilic and are primarily opportunistic in host-seeking behaviour. In 2005, the point prevalence of parasitaemia was estimated to be 16.3%: 46% due to *P*. *falciparum*, 39% due to *P*. *vivax*, and 11% due to mixed *P*. *falciparum*/*P*. *vivax* infections [17]. Local *P*. *vivax* strains have a typical equatorial relapse periodicity of 3–4 weeks [16,25].

Clinical trials conducted in Timika in 2004 and 2005 demonstrated failure of chloroquine and sulphadoxine + pyrimethamine for the treatment of uncomplicated falciparum malaria with a recurrence rate following combination treatment of 48% at day 28 [18]. Recurrence of *P*. *vivax* at day 28 post chloroquine monotherapy was even higher at 65% [18]. Ex vivo studies confirmed high-grade resistance to these drugs [19].

### Health facilities

Formal healthcare facilities in the district include Rumah Sakit Mitra Masyarakat (RSMM), a 110-bed hospital funded by a local mining company providing healthcare free of charge to indigenous Papuan communities and at a nominal cost to non-Papuan Indonesians. Additionally, there are 12 government-funded community primary health clinics (puskesmas) and 10 clinics administered by the local mining company. A government-funded hospital (Rumah Sakit Umum Daerah [RSUD]) opened at the end of 2008 but did not begin seeing substantial numbers of malaria patients until 2010. Antimalarial treatment can also be bought at a wide range of regulated and unregulated private sector clinics and facilities in Timika [23].

### Antimalarial treatment policy change

During the early 2000s, patients with uncomplicated malaria presenting to community clinics were treated with chloroquine plus sulphadoxine + pyrimethamine if they had falciparum malaria and with chloroquine plus low-dose primaquine (total dose 3.5 mg/kg) if they had vivax malaria. At the RSMM hospital, patients with uncomplicated malaria were treated with oral quinine, whereas patients with severe malaria received IV quinine. Those with *P*. *vivax* malaria also received unsupervised low-dose primaquine [16]. In March 2006, the policy for treatment of uncomplicated malaria due to any *Plasmodium* species at all public community clinics and the hospital was changed to DP, a regimen with a risk of *P*. *falciparum* recrudescence of 4.4% and *P*. *vivax* recurrence of 10% by day 42 [26]. At the same time, treatment of severe malaria was changed from IV quinine to IV artesunate [27], and the dose of unsupervised primaquine was doubled to 7 mg/kg divided over 14 days. The new unified antimalarial treatment policy was adopted by RSUD hospital upon opening. Healthcare providers disseminated information regarding the antimalarial policy change and the benefits of DP to clinics and to communities via village leaders.

### Community surveillance

All patients presenting to one of the formal sector community (puskesmas) clinics with symptoms consistent with malaria had capillary blood collected for blood film examination or had a rapid diagnostic test prior to antimalarial treatment. Between April 2004 and December 2009, weekly reports on the number of blood film examinations and the number of patients treated for malaria were collated by the district health authority. These reports were aggregated by the species of infection within four age bands: <1 year, 1 to 5 years, 5 to 10 years, and those older. Malariometric surveillance data were not collected from healthcare facilities in the private sector.

Cluster-randomized, cross-sectional surveys to determine treatment-seeking behaviour were conducted in 2005 and again in 2013, using an identical sampling strategy [17,23]. In 2005, during the pre–policy change period, 45.7% (349/764) of patients with malaria presented to public sector facilities and would have been detected by the surveillance network, but in 2013, 6 years after policy change, this figure had risen to 67.3% (66/98; *p* < 0.001) [28]. The shifts in treatment-seeking behaviour were apparent in all age groups. In the first household survey, 32.3% (10/31) of members who died of any cause within the preceding year did so at RSMM hospital compared to 26.3% (5/19) in 2013 (*p* = 0.656).

### Hospital surveillance

All patient presentations to RSMM between 2004 and 2013 (whether to the outpatients department, emergency department, or inpatient wards) were recorded by hospital administrators in a QPro database. Patients were identified using a unique hospital reference number. Demographic data and the clinical diagnoses assigned by the attending physician were collected. Drug prescriptions and results of full blood-count analyses from RSMM’s Coulter counter were recorded in separate databases and identified by the same individual hospital reference number. RSMM policy dictates that all outpatients with fever or other signs or symptoms consistent with malaria and all inpatients regardless of presentation have a thick film prepared for malaria microscopy. Thin films and rapid diagnostic tests were done after-hours when the laboratory was closed. At RSUD hospital, a malaria register was initiated in January 2010, documenting aggregated data on basic demographic details, infecting *Plasmodium* species, fulfilment of clinical criteria for severe malaria, admission status, and malaria-related death in patients at the hospital.

### Entomology and meteorology

Since 2002, vector-control activities in the region have been provided predominantly by the Public Health Malaria Control (PHMC) programme. These have included twice-yearly indoor residual spraying (IRS) and distribution of long-lasting insecticide-treated bed nets (LLINs) covering 10%–20% of households. Vector-control activities remained relatively constant from 2004 until mid-2013, when a large-scale IRS campaign and bed net distribution was commenced.

PHMC maintained entomological surveillance at five routine ‘sentinel’ sites representative of key locations in lowland Mimika from 1996 onwards. At each site, human-landing collections (HLCs) of mosquitoes were conducted at least once per week by 2 to 5 trained collectors for 5 to 10 hours during evening hours. Captured mosquitoes were examined and identified by microscopy and their species recorded based on key morphological characters. Data were combined to derive a human biting index over time. Automated daily rainfall was recorded at Kuala Kencana township, representing one of the 12 subdistricts in Mimika. Routine entomology and meteorological data were available from January 2004 until June 2009.

### Data preparation and statistical analysis

The analysis was conducted according to an a priori statistical plan (S1 Text) and is reported according to RECORD (S1 RECORD Checklist). Additional multivariable regression analyses, requested at statistical review, were also undertaken of key outcomes controlling for population size, vector biting, and monthly time trends. Data from the hospitals, community clinics, and meteorological and entomological surveillance were aggregated by calendar month. The surveillance period was divided into four periods: pre–policy change (April 2004 to March 2006), early transition (April 2006 to March 2008, an equal interval to that observed before policy change), late transition (April 2008 to December 2009, corresponding to the end of the community and entomology surveillance), and post transition (January 2010 to December 2013, corresponding to the period between the opening of the new RSUD hospital and the end of the study period) (S1 Fig, S1 Table).

Estimated malaria incidence rates were derived for the pre-policy and the early and late transition periods using absolute numbers of malaria cases from both the hospital and community surveillance, the estimated population at the time (assuming linear growth between the censuses), and estimates of the proportion of febrile patients seeking treatment within the malariometric surveillance system, obtained from the two household surveys in 2005 and 2013 (S1 Fig). In a sensitivity analysis, the incidence of malaria was also derived assuming no change in treatment-seeking behaviour. Analyses of malaria-related morbidity and mortality were limited to RSMM data, as the necessary information was not collected from the other sites. To account for monthly time trends, vector biting, and population growth, Poisson regression analyses of the data pre-2009 were performed to estimate the adjusted incidence rate ratios (IRRs) for falciparum and for vivax malaria for the late transition period versus the pre–policy change period. Similar analyses were undertaken for hospital admissions using binomial regression to estimate the risk ratio.

All graphing and statistical analysis was done in STATA version 15.1 (StataCorp, College Station, TX, United States). Temporal trends in outcomes were presented graphically over the entire study period. Comparisons of outcomes before and after policy change were made using medians (with interquartile ranges [IQRs]), proportions (*n*/*N* with absolute differences and binomial 95% confidence intervals [CIs]), incidence rates (per 1,000 person-years [py] with IRRs and Poisson 95% CIs). Comparisons of prospectively collected community and hospital surveillance data were restricted to the pre-policy and late transition periods to ensure inclusion of the community surveillance (which ended in December 2009) and to avoid bias associated with the opening of RSUD (which began admitting significant numbers of malaria patients in January 2010). *p*-Values were not presented, since numbers of cases from the community clinics and hospitals were large and statistical significance was achieved even in the absence of clinical significance.

### Ethical approval

Ethical approval for this study was obtained from the Health Research Ethics Committees of the University of Gadjah Mada, Indonesia (KE/FK/544/EC), and Menzies School of Health Research, Darwin, Australia (HREC 10.1397).

## Results

### Rainfall and entomology

**Fig 2:**
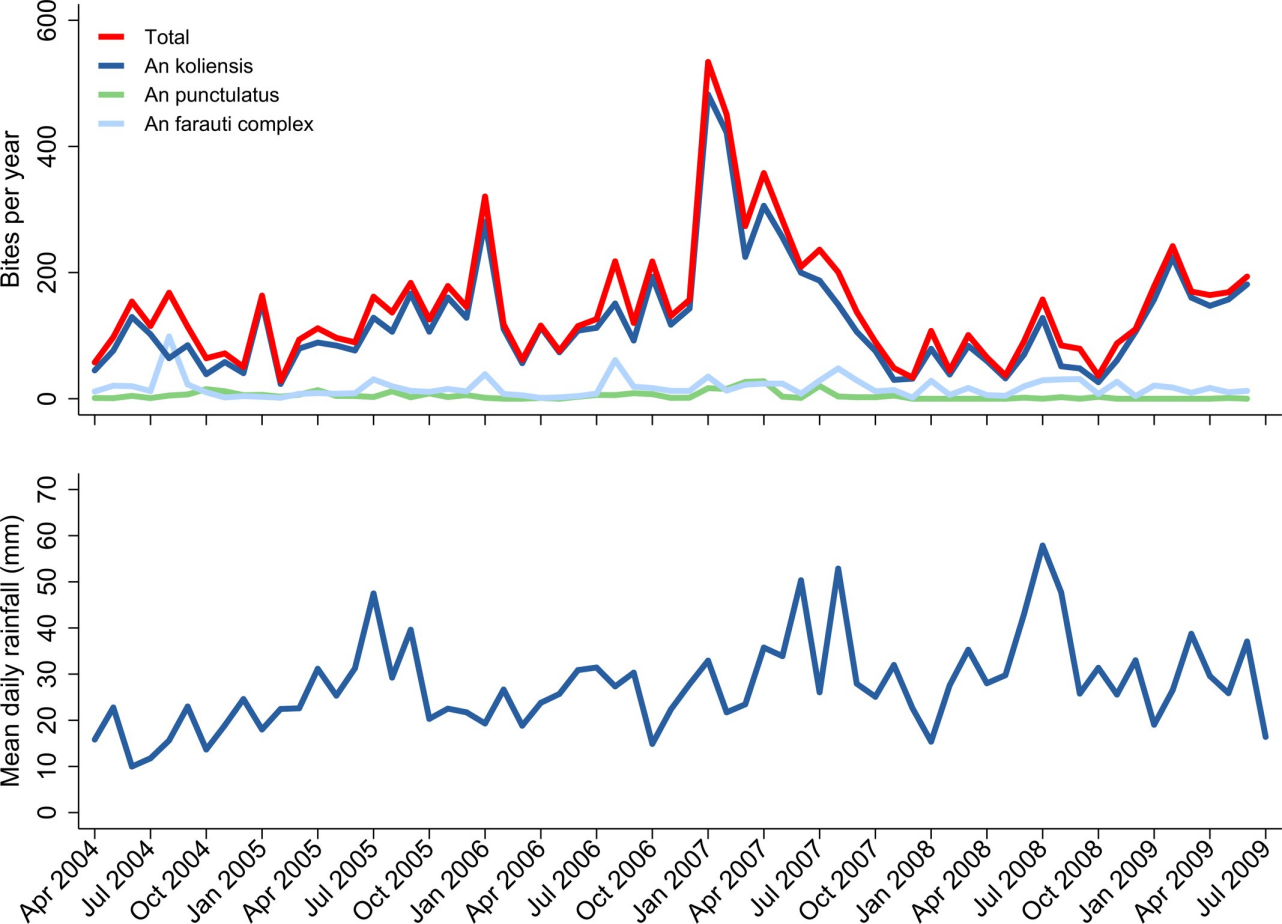
Community entomological and meteorological surveillance. Predicted number of *Anopheles* (‘An’) mosquito bites per year by month for the three main *Plasmodium* vectors in Mimika District (top) and mean daily rainfall by month (bottom).

Between April 2004 and June 2009, the mean daily rainfall was 27.8 mm with small peaks in mid-2005, mid-2007, and mid-2008 (Fig 2, S1 Data). Over the same period, 204,968 human-hours of nighttime mosquito collections were conducted; the estimated mean number of anopheline bites per py was 145 (range 28 to 534) (Fig 2, S1 Data). There was a large peak in the number of mosquitoes caught throughout much of 2007; the mean estimated number of bites per py during this period was 238 (range 34 to 534). Overall, *A*. *koliensis* accounted for 85.0% (6,992/8,222) of mosquitoes captured by HLC, compared to 11.6% (956/8,222) for *A*. *farauti* species complex and 3.3% (274/8,222) for *A*. *punctulatus*.

### Community facilities

**Fig 3:**
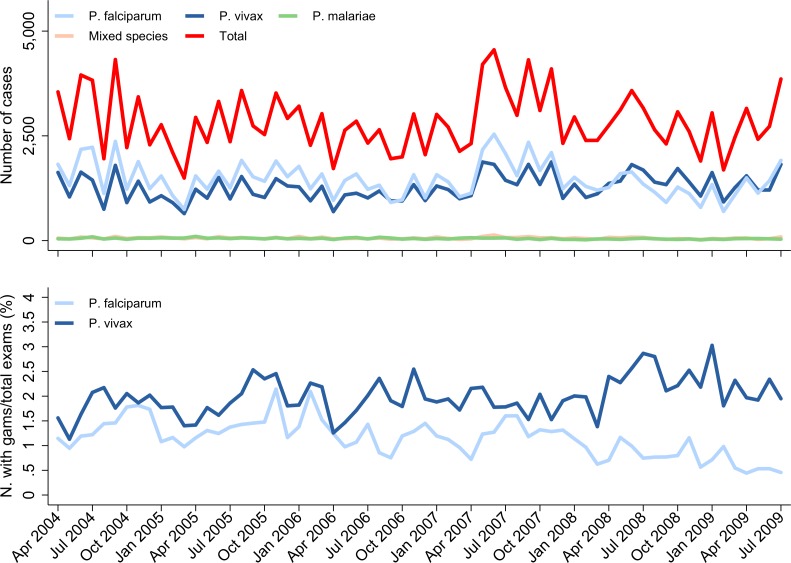
Malaria surveillance gathered from community healthcare facilities. Absolute number (‘N.’) of malaria cases detected within the community surveillance network by *Plasmodium* species and month (top) and proportion of all blood films examined in the community with gametocytes (‘gams’) by *Plasmodium* species and time (bottom).

Data were gathered from 12 community outpatient facilities over a period of 69 months (April 2004 to December 2009). A total of 671,386 blood films were examined, of which 193,566 (28.8%) were positive for malaria; 98,530 (50.9%) were due to *P*. *falciparum*, 87,632 (45.3%) were due to *P*. *vivax*, 3,308 (1.7%) were due to *P*. *malariae*, and 4,096 (2.1%) were mixed species infections (Table 1). Slide positivity for *P*. *falciparum* declined from 14.8% (37,304/251,286) before treatment policy change to 13.0% (25,362/194,792) in the late transition period, a difference of −1.8% (95% CI −2.0% to −1.6%). Over the same period, the slide positivity for *P*. *vivax* increased from 11.5% (28,872/251,286) to 14.8% (28,861/194,792), difference = 3.3% (95% CI 3.1%–3.5%). The corresponding proportion of malaria infections due to *P*. *vivax* rose from 44.1% (30,444/69,098) to 53.3% (29,934/56,125), difference = 9.2% (95% CI 8.7%–9.8%) (Fig 3, S2 Data). In multivariable analyses controlling for population size, vector biting, and monthly time trends, the IRR for the late transition period compared with the pre–policy change period was 0.70 (95% CI 0.67–0.74) for falciparum malaria and 1.02 (95% CI 0.97–1.07) for vivax malaria.

**Table 1:** Patients presenting and diagnosed with malaria at community clinics and the RSMM hospital.

	Pre–Policy Change Period	Early Transition Period	Late Transition Period	Posttransition Period	Total
	Apr 2004–Mar 2006	Apr 2006–Mar 2008	Apr 2008–Dec 2009	Jan 2010–Dec 2013	
**Community Surveillance**					
Total slide examinations	251,286	225,308	194,792	**-**	**671,386**
*P*. *falciparum*	37,304 (14.8%)	35,864 (15.9%)	25,362 (13.0%)	**-**	98,530
*P*. *vivax*	28,872 (11.5%)	29,899 (13.3%)	28,861 (14.8%)	**-**	87,632
*P*. *malariae*	1,350 (0.5%)	1,129 (0.5%)	829 (0.4%)	**-**	3,308
Mixed species	1,572 (0.6%)	1,451 (0.6%)	1,073 (0.6%)	**-**	4,096
Total malaria cases	69,098 (27.5%)	68,343 (30.3%)	56,125 (28.8%)	**-**	193,566
*P*. *falciparum* gametocytes	3,491 (1.4%)	2,636 (1.2%)	1,419 (0.7%)	**-**	7,546
*P*. *vivax* gametocytes	4,700 (1.9%)	4,192 (1.9%)	4,403 (2.3%)	**-**	13,295
**Outpatient presentations to the RSMM Hospital**					
Total outpatient presentations	155,029	181,493	169,391	453,506	959,419
*P*. *falciparum*	13,654 (8.8%)	18,977 (10.5%)	14,801 (8.7%)	35,259 (7.8%)	82,691
*P*. *vivax*	6,056 (3.9%)	9,472 (5.2%)	11,198 (6.6%)	32,464 (7.2%)	59,190
*P*. *malariae*	411 (0.3%)	871 (0.5%)	1,230 (0.7%)	2,153 (0.5%)	4,665
*P*. *ovale*	17 (0.01%)	25 (0.01%)	36 (0.02%)	32 (0.01%)	110
Mixed species	906 (0.6%)	2,248 (1.2%)	2,066 (1.2%)	16,614 (3.7%)	21,834
All malaria	21,044 (13.6%)	31,593 (17.4%)	29,331 (17.3%)	86,522 (19.1%)	168,490
**Hospital admissions at the RSMM Hospital**					
Total hospital admissions	19,260	20,293	16,921	38,781	95,255
*P*. *falciparum*	5,311 (27.6%)	4,863 (24%)	2,890 (17.1%)	4,323 (11.1%)	17,387
*P*. *vivax*	1,489 (7.7%)	1,383 (6.8%)	1,199 (7.1%)	2,045 (5.3%)	6,116
*P*. *malariae*	70 (0.4%)	105 (0.5%)	122 (0.7%)	135 (0.3%)	432
*P*. *ovale*	1	3	3	3	10
Mixed species	874 (4.5%)	720 (3.5%)	572 (3.4%)	1,779 (4.6%)	3,945
All malaria	7,745 (40.2%)	7,074 (34.9%)	4,786 (28.3%)	8,285 (21.4%)	27,890

Abbreviation: RSMM, Rumah Sakit Mitra Masyarakat.

The proportion of all blood films read that were positive for *P*. *falciparum* gametocytes fell steadily over the study period from 1.4% (3,491/251,286) pre–policy change to 0.7% (1,419/194,792) in the late transition period, a difference of −0.7% (95% CI −0.7% to −0.6%) (Fig 3). Over the same time interval, there was an increase in the overall gametocyte slide positivity for *P*. *vivax*, which rose from 1.9% (4,700/251,286) to 2.3% (4,403/194,792), a difference of 0.4% (95% CI 0.3%–0.5%).

### RSMM hospital data

Data from RSMM were available for 117 months (April 2004 to December 2013). Overall, there were 1,054,674 patient presentations to the hospital, of which 196,380 (18.6%) were associated with malaria, 100,078 (51.0%) with *P*. *falciparum*, 65,306 (33.3%) with *P*. *vivax*, 5,097 (2.6%) with *P*. *malariae*, 120 (0.06%) with *P*. *ovale*, and 25,779 (13.1%) with mixed species infections. In total, 27,890 (14.2%) of the patients with malaria required admission to hospital, and 595 (0.3%) died in hospital. In the posttransition period, 22.5% (27,480/122,232) of malaria diagnosed at a tertiary facility was at the newly opened RSUD hospital (S3 Data); therefore, before-and-after comparisons at the RSMM hospital were only made between the pre–policy change era and the late transition period.

**Fig 4:**
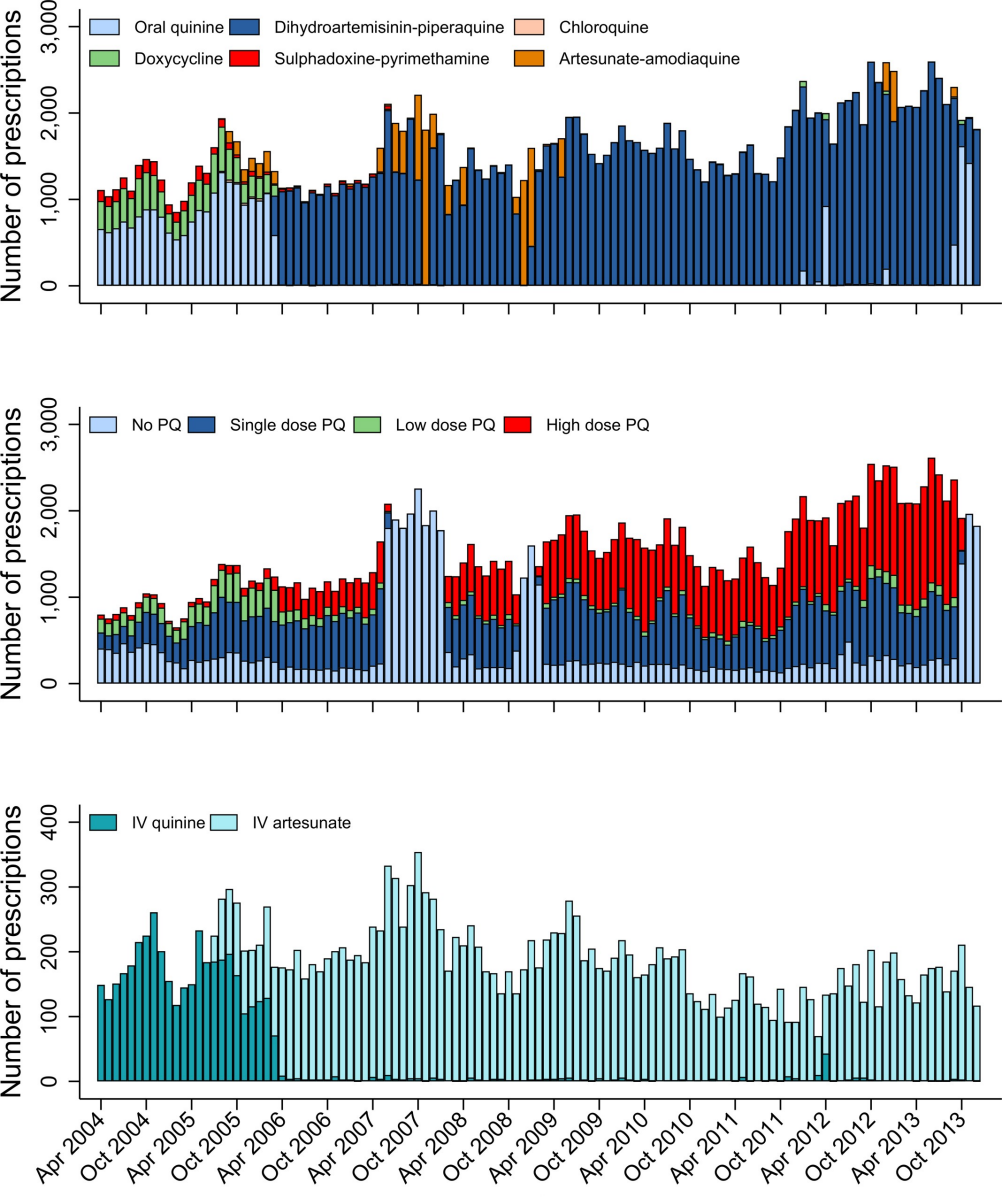
Antimalarial drug prescriptions at Mitra Masyarakat hospital. Number of oral blood schizontocidal drug prescriptions (top), PQ prescriptions (middle), and IV blood schizontocidal drug prescriptions (bottom) at Mitra Masyarakat Hospital by month. Low-dose PQ is defined as a total dose of ≥1.5 to <5 mg/kg. High-dose PQ is defined as a total dose of ≥5 mg/kg. IV, intravenous; PQ, primaquine.

Before policy change, the most commonly prescribed blood schizontocide at RSMM hospital was oral quinine (20,364/24,538; 83.0%) (Fig 4); thereafter, DP was prescribed in 88.6% (139,002/156,902) and artesunate-amodiaquine in 6.0% (9,442/156,902) of malaria cases. Patients requiring IV therapy were prescribed quinine in 83.1% (3,858/4,641) of cases before policy change, but thereafter, 98.5% (16,414/16,661) were treated with IV artesunate. Before policy change, 63.5% (4,455/7,019) of patients with *P*. *vivax* malaria were treated with a 14-day primaquine regimen, and after policy change, this rose to 71.1% (51,344/72,196).

**Fig 5:**
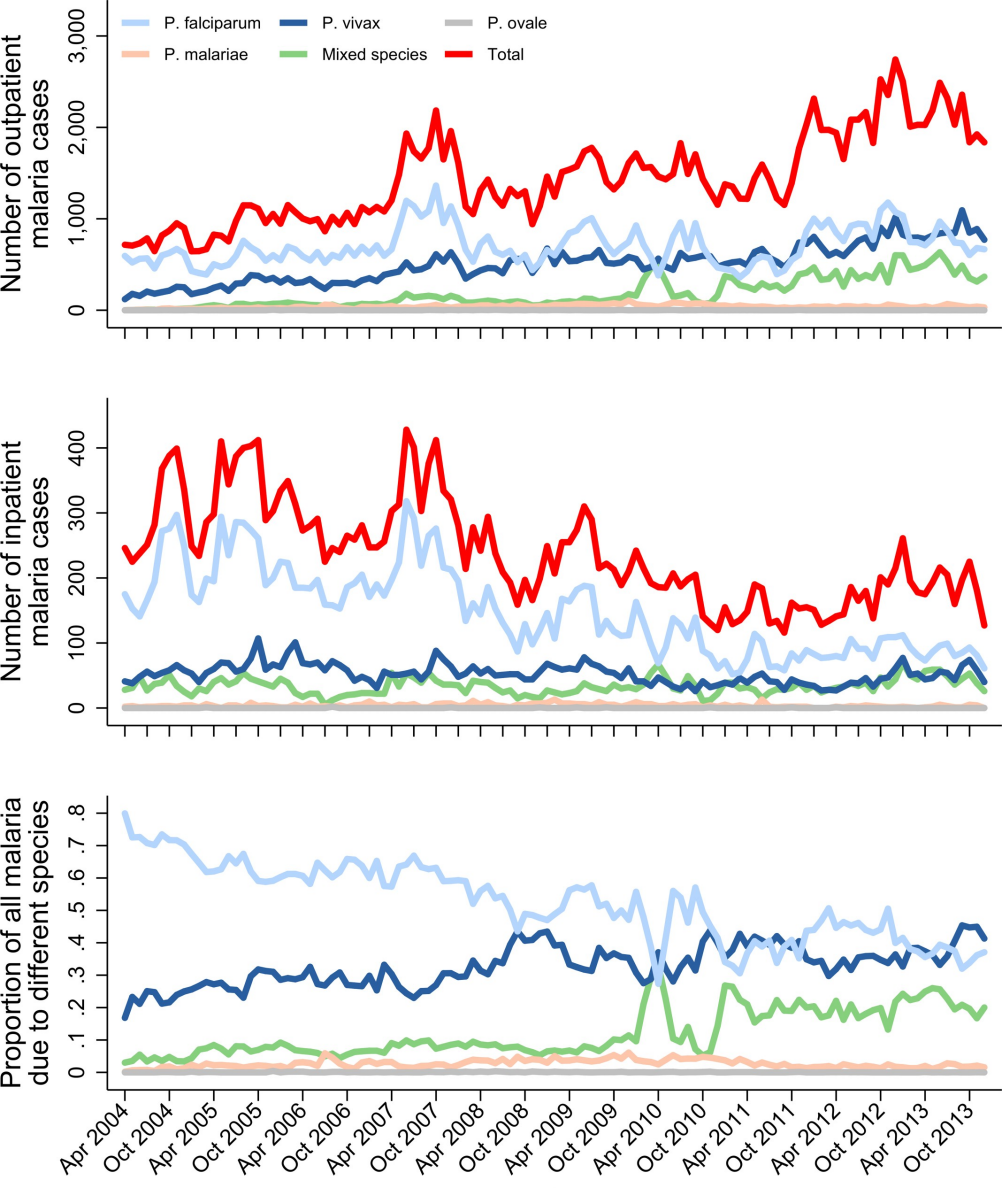
Malaria cases at Mitra Masyarakat hospital. Number of outpatient (top) and inpatient (middle) malaria cases at Mitra Masyarakat Hospital by *Plasmodium* species and month and proportion of all hospital malaria cases due to the different *Plasmodium* species (bottom).

**Fig 6:**
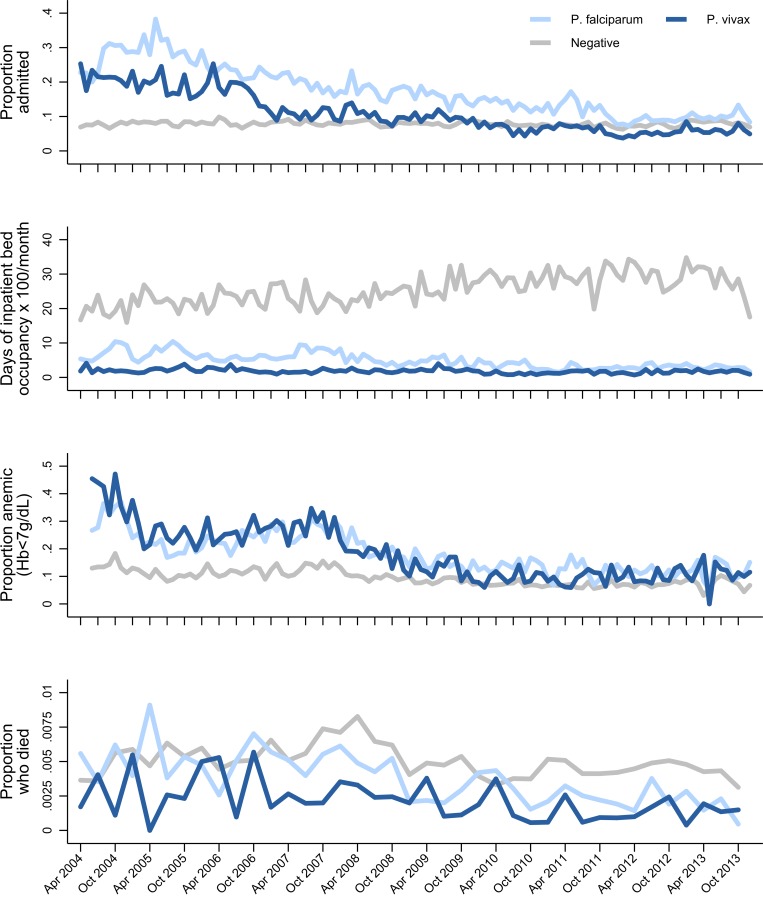
Malaria-related morbidity and mortality at Mitra Masyarakat hospital. Proportion of all patients presenting to Mitra Masyarakat Hospital who were admitted by *Plasmodium* species and month (top). Total number of days of inpatient bed occupancy × 100/month by *Plasmodium* species (second from top). Proportion of all patients presenting to Mitra Masyarakat Hospital who were anaemic (Hb < 7 g/dL) (third from top) and who died (bottom) by *Plasmodium* species and month. Hb, haemoglobin.

The proportion of all presentations to RSMM that were related to malaria rose from 16.5% (28,789/174,289) before policy change to 18.3% (34,117/186,312) in the late transition period (difference of 1.8% [95% CI 1.5%–2.0%]) (Fig 5). This was driven by a rise in the proportion of outpatients with malaria, which increased from 13.6% (21,044/155,029) to 17.3% (29,331/169,391), a difference of 3.7% (95% CI 3.5%–4.0%). Over the same period, the proportion of inpatients with malaria fell from 40.2% (7,745/19,260) to 28.3% (4,786/16,921; difference of −11.9% [95% CI −12.9% to −11.0%]), and the proportion of patients with malaria requiring admission fell from 26.9% (7,745/28,789) to 14.0% (4,786/34,117; difference of −12.9% [95% CI −13.5% to −12.2%]) (Fig 6, Table 1 and S3 Data). In multivariable analyses comparing the pre–policy change and late transition period, after controlling for monthly trends, the proportion of inpatients with malaria decreased by 0.56-fold (95% CI 0.51–0.60), and the proportion of patients with malaria requiring admission fell by 0.82-fold (95% CI 0.75–0.90).

**Fig 7:**
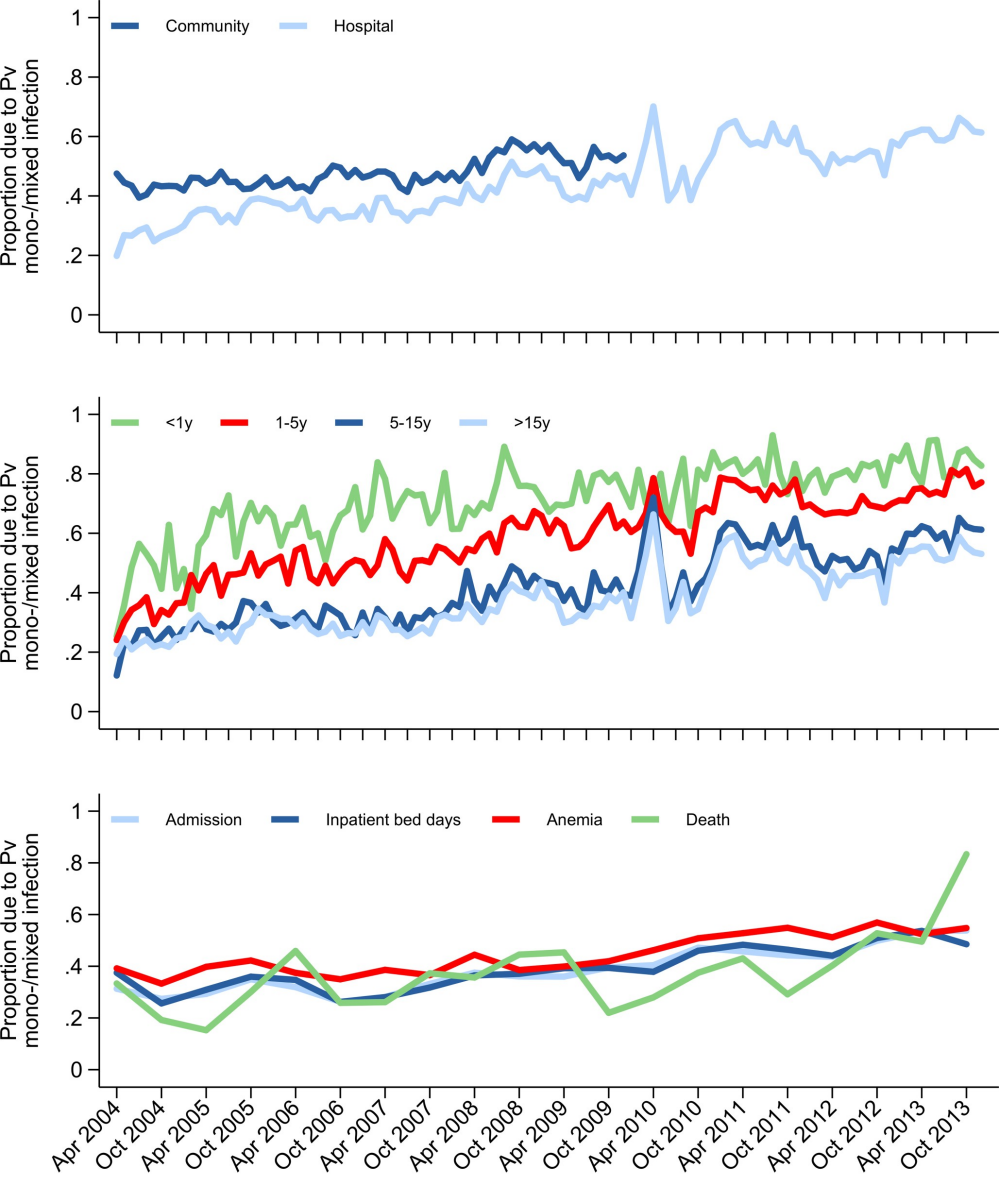
Proportion of malaria morbidity and mortality attributable to Pv mono- or mixed infection. In the community and hospital (top). In the hospital stratified by age group (middle). In the hospital: malaria-related hospital admissions, inpatient bed days, malaria-related anaemia, and malaria-related deaths (bottom). Pv, *P*. *vivax*.

Of the patients admitted with malaria, the median length of stay decreased from 3 days (IQR 2–4) to 2 days (IQR 2–4), and this was associated with a fall in the median total monthly inpatient bed occupancy due to malaria from 1,033.5 days (IQR 897–1,251.5) pre–policy change to 769.5 days (IQR 685.5–856) post policy change (Fig 6). Overall, the proportion of malaria cases due to *P*. *vivax* mono- or mixed species infection rose from 32.4% (9,325/28,789) to 44.1% (15,035/34,117), a difference of 11.7% (95% CI 10.9%–12.4%) (Fig 7). Malaria due to any *Plasmodium* species collectively accounted for 57.7% (4,388/7,603) of all severe anaemia at the hospital before policy change and 41.7% (1,928/4,627) in the late transition period, a difference of −16.0% (95% CI −17.8% to −14.2%) (Fig 7).

### Malaria-attributable mortality

Before policy change, malaria accounted for 15.5% (137/886) of all deaths at RSMM, with 0.48% (137/28,789) of patients who presented with malaria dying during their hospital encounter. In the late transition period, the corresponding figures were 10.4% (100/961; difference = −5.1% [95% CI −8.1 to −2.0]) and 0.29% (100/34,117; difference = −0.18% [95% CI −0.28 to −0.08]) (Fig 6, S3 Data). Over the same period, there was a significant fall in the mortality attributable to *P*. *falciparum* (0.53% [100/18,965] versus 0.32% [57/17,691]; difference = −0.21% [95% CI −0.34 to −0.07]), but this was not apparent for *P*. *vivax* (0.28% [21/7,545] versus 0.23% [28/12,397]; difference = −0.05% [95% CI −0.20 to 0.09]) or nonmalarial disease (0.51% [749/145,500] versus 0.57% [861/152,195], difference = 0.05% [95% CI −0.002 to 0.10]). Overall, *P*. *vivax* accounted for 15.3% (21/137) of malaria-related deaths pre–policy change and 28.0% (28/100) in the late transition period, a difference of 12.7% (95% CI 2.0%–23.3%) (S3 Data).

Assuming that 30% of deaths in the region occurred at RSMM hospital, the overall malaria-attributable mortality rate in the population of Mimika District fell from 1.90 (95% CI 1.73–2.08) per thousand py before the policy change to 1.29 (95% CI 1.15–1.43) per 1,000 py in the late transition period, a rate difference of −0.61 (95% CI −0.83 to −0.39) per 1,000 py.

### Combined estimates of malaria incidence

When community and hospital surveillance data were combined, a total of 295,971 cases of malaria were diagnosed between April 2004 and December 2009. The overall incidence of malaria was 406 (95% CI 404–409) per 1,000 py before policy change, 372 (95% CI 370–374) per 1,000 py in the early transition period, and 351 (95% CI 349–354) per 1,000 py in the late transition period (Table 2, S4 Data). Assuming that 45.7% of patients with malaria were detected by the surveillance network pre–policy change and 67.3% in the late and posttransition periods, the overall incidence of *P*. *falciparum* fell from 511 to 249 per 1,000 py (IRR = 0.49 [95% CI 0.48–0.49]), whereas the incidence of *P*. *vivax* fell from 331 to 239 per 1,000 py (IRR = 0.72 [95% CI 0.71–0.73]). In a sensitivity analysis assuming no shift in treatment-seeking behaviour, the incidence of *P*. *falciparum* fell from 234 to 168 per 1,000 py (IRR = 0.72 [95% CI 0.71–0.73]), whereas the incidence of *P*. *vivax* rose from 152 to 161 per 1,000 py (IRR = 1.06 [95% CI 1.05–1.08]).

**Table 2:** Total number of community and hospital malaria cases and incidence of malaria before and after policy change.

	Pre–Policy Change Period	Early Transition Period	Late Transition Period	Posttransition Period
	Apr 2004–Mar 2006	Apr 2006–Mar 2008	Apr 2008–Dec 2009	Jan 2010–Dec 2013
Population of Mimika^a^	120,457	143,723	148,124	189,447
Months of observation	24	24	21	48
Person-years of observation	240,914	287,446	259,217	757,788
Percent of patients with malaria attending the surveillance network^b^	45.7%	-	-	67.3%
**Number of malaria cases**				
*P*. *falciparum*	56,269	59,704	43,464	53,749^c^
*P*. *vivax*	36,417	40,754	41,659	46,491^c^
*P*. *malariae*	1,831	2,105	2,193	2,477^c^
Mixed species	3,352	4,419	3,719	19,515^c^
Overall^d^	97,887	107,010	91,074	122,267^c^
**Incidence rate of malaria (per 1,000 person-years) assuming complete ascertainment of all malaria patients (95% confidence interval)**				
*P*. *falciparum*	234 (232–236)	208 (206–209)	168 (166–169)	-
*P*. *vivax*	152 (150–153)	142 (140–143)	161 (159–162)	-
*P*. *malariae*	7.6 (7.3–8.0)	7.3 (7.0–7.6)	8.5 (8.1–8.8)	-
Mixed species	13.9 (13.4–14.4)	15.4 (14.9–15.8)	14.3 (13.9–14.8)	-
Overall	406 (404–409)	372 (370–375)	351 (349–354)	-
**Incidence rate of malaria (per 1,000 person-years) assuming shifts in treatment-seeking behaviour (95% confidence interval)**				
*P*. *falciparum*	511 (508–514)	-	249 (247–251)	-
*P*. *vivax*	331 (328–333)	-	239 (237–241)	-
*P*. *malariae*	16.6 (16.1–17.2)	-	12.6 (12.1–13.0)	-
Mixed species	30.4 (29.8–31.2)	-	21.3 (20.8–21.9)	-
Overall	889 (885–893)	-	522 (519–525)	*-*

^a^Population estimates taken at midpoint of the time interval.

^b^Estimates for the proportion of patients with malaria attending the public provider and thus who will have been detected by the surveillance network were derived from household surveys conducted in 2005 and 2013, reported previously [28].

^c^Includes patients attending the new RSUD hospital but excludes community cases due to surveillance finishing in December 2009.

^d^Includes 120 cases of *P*. *ovale*.

Abbreviation: RSUD, Rumah Sakit Umum Daerah.

## Discussion

In March 2006, Indonesia was the first malaria-endemic country to adopt a unified schizontocidal treatment policy for malaria due to any *Plasmodium* species: DP for uncomplicated malaria and IV artesunate for severe malaria. These changes came on a background of failing treatment regimens due to high-grade multidrug resistance in both *P*. *falciparum* and *P*. *vivax* species. In Mimika District, southern Papua, the uptake of the new policy was rapid, with the new treatment regimens adopted into practice in most public health facilities within a month. In this high-transmission setting, we found that the implementation of highly effective antimalarial treatment regimens was associated with a marked reduction in both malaria-related morbidity and mortality. The incidence of malaria and the proportion of malaria requiring admission to hospital fell by one-half, bed occupancy of patients with malaria fell by 26%, and malaria-related mortality fell by one-third. Associated with these changes, the proportion of patients with malaria attributable to *P*. *vivax* increased from 41% to 54%, and the proportion of malaria-related deaths attributable to *P*. *vivax* rose from 15% to 28%.

Our study highlights the complexity of defining temporal changes in the burden of malaria at a population level over a long period of time [1,2,29]. During the 9 years of surveillance, there was a substantial increase in the local population, fluctuations in rainfall and vector mosquito numbers, and marked changes in healthcare provision and treatment-seeking behaviour. Our comprehensive surveillance quantified patient numbers at the only lowland hospital (until late 2009) and all public and mine-supported clinics, which collectively diagnosed and treated almost half a million cases of malaria over the study period. Although these clinics offered healthcare for free or at a nominal cost, our treatment-seeking surveys suggested that initially only 46% of patients with malaria sought treatment in the public sector. However, following the change in policy, there was a significant shift in behaviour, with 67% of patients seeking treatment in the public sector where they could access DP, a drug perceived to be highly effective compared to previously available treatments [28]. This shift in behaviour, along with the rise in the total population and an increase in vector numbers in 2007 (Fig 2), may have contributed to the initial surge in malaria cases and slide positivity observed in the community and hospital outpatients department in the early transition period but a subsequent fall in these metrics in the late transition period as the impact of ACT on *P*. *falciparum* began to manifest (Fig 3). Conversely, in the latter part of the study, a new public hospital facility (RSUD) outside of the initial surveillance network was opened and assumed care of approximately 20% of malaria inpatients, acting to artificially decrease the burden of malaria at RSMM hospital. To control for these confounding factors, the overall temporal trends by month are presented, but the comparisons pre–and post–policy change were restricted conservatively to the period immediately before policy change and the late transition period, prior to the opening of the new hospital facility.

The replacement of failing treatment regimens with highly efficacious schizontocidal treatment has potential to reduce malaria-related morbidity and mortality and decrease the risk of recurrent parasitaemia and ongoing transmission [30]. In Papua in 2005, the efficacy of DP against *P*. *falciparum* and *P*. *vivax* was greater than 95%, with antimalarial efficacy sustained against both species throughout the study period [26,31]. In the same year, a clinical trial of patients with severe malaria at the RSMM hospital demonstrated that IV artesunate reduced associated mortality by 35% compared to IV quinine [27]. Our analysis highlights that, following implementation of both of these artemisinin-based treatment strategies in April 2006, there was a significant reduction in malaria-related morbidity and mortality, which was most apparent at the RSMM hospital. Whilst outpatient numbers actually increased over the study period, both the absolute number of malaria admissions and the proportion of malaria patients requiring admission fell (the latter from 27% to 14%). This was associated with a marked reduction in total bed occupancy due to malaria, shorter admission times, and a lower risk of severe anaemia- and malaria-related mortality. In total, 264 bed days per month were made available at the hospital for the treatment of other diseases. These findings are consistent with African studies that have shown that the most prominent impact of enhanced malaria control activities is a reduction in severe malaria and mortality [32,33]. The variation in malaria morbidity was less marked in the community, where absolute numbers of patients remained high and where there was only a modest fall in malaria prevalence from 16.3% to 12.2%. However, after accounting for population growth and shifts in treatment-seeking behaviour, the estimated overall incidence of malaria in the community also fell significantly.

A consistent finding in both the hospital and community setting was the marked increase in the proportion of malaria caused by *P*. *vivax*. Policy change was associated with a differential variation between species in the overall cases of malaria, severe disease, and gametocyte carriage. At the start of the study, *P*. *vivax* accounted for 32% of all malaria at the hospital and 44% in the community. By the late transition period, *P*. *vivax* was the predominant cause of malaria, accounting for 54% of all malaria cases. Whereas the overall risk of mortality in patients presenting with *P*. *falciparum* fell from 0.53% to less than 0.25% in the posttransition period, there was no fall in mortality associated with *P*. *vivax*, possibly reflecting a higher likelihood of concomitant nonmalarial morbidities in severely ill patients with *P*. *vivax* malaria [34,35]. In the community, the proportion of patients with *P*. *falciparum* gametocytes on blood film examination halved (from 1.4% to 0.7%), whereas the proportion of patients with *P*. *vivax* gametocytes remained unchanged at about 2% (Fig 3).

*P*. *vivax* is less amenable than *P*. *falciparum* to control by enhanced or scaled-up antimalarial treatment efforts [6,7,36]. Mass drug administration that does not include antirelapse therapy has little effect on *P*. *vivax* [3]. There are several biological reasons for this refractoriness. Firstly, *P*. *vivax* gametocytes appear early during the course of an infection and are therefore more likely than *P*. *falciparum* gametocytes, which appear later, to have been transmitted to mosquitoes prior to antimalarial treatment. Secondly, failure to sterilize the liver of hypnozoites can result in multiple subsequent relapses and thus much greater transmission potential from a single inoculation of *P*. *vivax* as compared with *P*. *falciparum*. Thirdly, immunity to *P*. *vivax* develops early in endemic regions because of the high force of infection from relapses [22,37]. This results in a large pool of asymptomatic patients who probably still harbour gametocytes and therefore remain infectious to mosquitoes [38,39].

Although DP provides posttreatment prophylaxis against *P*. *vivax* recurrence for up to 42 days, it has minimal effect on *P*. *vivax* relapses thereafter. At the same time as the change in blood schizontocidal treatment policy, the recommended dose of primaquine for radical cure of *P*. *vivax* infections was revised from a total dose of 3.5 mg/kg to 7 mg/kg to treat the relatively primaquine-tolerant *P*. *vivax* strains in Papua [40]. If safely and effectively delivered, high-dose primaquine regimens should produce a significant reduction in *P*. *vivax* transmission. However, in Timika, the provision of unsupervised high-dose primaquine combined with DP has, at best, a modest effect on the likelihood of representation to hospital with vivax malaria [16]. We postulate that nonadherence to unsupervised primaquine is one of the most likely explanations for the minimal decline in *P*. *vivax* incidence in the region.

Our study has several important strengths. The multifaceted surveillance system incorporated prospectively collected community and hospital data along with before-and-after cross-sectional surveys, providing a means of checking the consistency of the findings across various settings and thus increasing confidence in the internal validity of our results. Data on nonmalaria presentations to hospital enabled us to control the hospital data for population growth by presenting the proportional burden of malaria over time. High-quality microscopy services both at the hospital and in the community clinics are maintained by accredited microscopists, whose performance is reviewed regularly [22]. Consistent procedures for examination and reporting of blood films therefore support the validity of longitudinal assessments of *Plasmodium* species distributions observed in our analysis. Individualized clinical data at RSMM with linkage to pharmacy and haematology data enabled a very large-scale assessment of the temporal trends in malaria-related morbidity (in addition to incidence) in the hospital population.

Our study also has some significant limitations. Estimates of the local population were imprecise because of large transient migrant groups who were excluded from censuses. This was an issue particularly in the latter years, when the population at risk of malaria may have been underestimated; thus, our estimates of the reduction in malaria incidence are likely to be conservative. The estimated change in the proportion of malaria patients detected by the surveillance network was derived from two assessments of treatment-seeking behaviour in 2005 and 2013 [28]. In a sensitivity analysis, assuming no shift in treatment-seeking behaviour, the estimated reduction in the incidence of falciparum malaria was only 28%, with a 6% rise in the incidence *P*. *vivax* malaria. However, changes in treatment-seeking behaviour should not have confounded estimates of the proportion of malaria due to *P*. *vivax* or the reduction in hospital-related morbidity. The surveillance system captured changes in human–mosquito attack rates and climate variability but did not record concurrent vector-control interventions. Throughout most of the study period, formal vector-control activities were supported by the mine-supported PHMC program. These activities remained relatively constant, apart from a large bed net distribution and IRS program in urban Timika that commenced in 2013, towards the end of the posttransition period. Changes in vector-control measures were therefore unlikely to have influenced to a significant extent the reduction in malaria incidence between the pre–policy change and late transition periods. Finally, in view of the variations in treatment-seeking behaviour and healthcare provision, an a priori decision was made to compare the malarial outcomes between the pre-policy and the late transition periods. Although comparison of dichotomised temporal data can result in loss of power and in type I error, multivariable regression analyses of unconstrained monthly data confirmed the observed trends and demonstrated that they were still apparent after controlling for population growth and vector biting.

In summary, in Papua, Indonesia, a change in antimalarial treatment policy from failing drugs to highly efficacious artemisinin-based treatment for both uncomplicated and severe malaria was associated with a modest reduction in the overall incidence of malaria but a significant reduction in malaria-related hospital admissions and mortality. In this area coendemic for both *P*. *falciparum* and *P*. *vivax*, there was a marked increase in the proportion of malaria attributable to *P*. *vivax*. Additional scale-up of the existing treatment strategy is likely to result in further reductions in *P*. *falciparum* transmission; however, in order to reduce *P*. *vivax* transmission significantly, antirelapse therapy will need to be delivered more effectively. Novel strategies are being developed to improve primaquine adherence through community education campaigns and directly observed supervision of treatment [41]. The availability of point-of-care testing for G6PD deficiency raises the possibility of introducing short-course high-daily-dose primaquine regimens and tafenoquine that will facilitate further adherence to a complete course of treatment [42,43]. In coendemic regions, access to safe and effective radical cure of malaria for all patients at risk of malaria will be critical for the timely elimination of malaria.

## Supporting information

S1 RECORD Checklist (DOCX)

S1 Text Statistical analysis plan. (DOCX)

S1 Fig Timelines for different components of the malaria surveillance system. First and second household surveys were reported previously [28].
(TIF)

S1 Table List of healthcare facilities within the Mimika District and the periods for which data were collected for malaria surveillance. (DOCX)

S1 Data Entomology and rainfall data (April 2004 and July 2009). (XLSX)

S2 Data Data collected from the community surveillance (April 2004 and December 2009). (XLSX)

S3 Data Data collected from patients presenting to the RSMM hospital inpatient and outpatient departments (April 2004 and December 2013). RSMM, Rumah Sakit Mitra Masyarakat.
(XLSX)

S4 Data Combined data and derived variables from patients presenting to the community, RSMM hospital, and RSUD hospital. RSMM, Rumah Sakit Mitra Masyarakat; RSUD, Rumah Sakit Umum Daerah.
(XLSX)

## Abbreviations

ACT: artemisinin combination therapy
CI: confidence interval
DP: dihydroartemisinin plus piperaquine
G6PD: glucose-6-phosphate dehydrogenase
HLC: human-landing collection
IQR: interquartile range
IRR: incidence rate ratio
IRS: indoor residual spraying
IV: intravenous
LLIN: long-lasting insecticide-treated bed net
PHMC: Public Health Malaria Control
py: person-years
RSMM: Rumah Sakit Mitra Masyarakat
RSUD: Rumah Sakit Umum Daerah
