# Supplementary material for: Malaria morbidity and mortality following introduction of a universal policy of artemisinin-based treatment for malaria in Papua, Indonesia: A longitudinal surveillance study
Source: PLoS Med. 2019 May 29;16(5):e1002815. doi: 10.1371/journal.pmed.1002815 (PMC6541239; doi:10.1371/journal.pmed.1002815)
Supplement: S1 Text — (DOCX) [file pmed.1002815.s002.docx]

Statistical Analysis Plan

**Determining the Impact of Introducing a Universal Policy of Artemisinin-based Treatment for Malaria Due to Any *Plasmodium* Species in Timika, Papua**

**Contents**

[A. Introduction 1](#_Toc531785536)

[B. Outcomes of interest 1](#_Toc531785537)

[1. Community 1](#_Toc531785538)

[2. Hospital 2](#_Toc531785539)

[3. Combined community and hospital 2](#_Toc531785540)

[C. Available Data 2](#_Toc531785541)

[1. Community 2](#_Toc531785542)

[2. Hospital 3](#_Toc531785543)

[D. Major potential sources of confounding 3](#_Toc531785544)

[E. Analysis Plan 4](#_Toc531785545)

[1. Graphical presentations 4](#_Toc531785546)

[a. Community 4](#_Toc531785547)

[b. Hospital 4](#_Toc531785548)

[2. Statistical comparisons 5](#_Toc531785549)

[F. Means of controlling for potential confounding factors 5](#_Toc531785550)

# Introduction

Clinical and *in vitro* research work during the early 2000s identified severe multidrug resistance amongst *Plasmodium falciparum* and *P. vivax* strains in Timika, Papua, Indonesia. Separate, multicentre studies during the same period demonstrated the superiority of intravenous artesunate over quinine for the treatment of severe malaria. In response, Indonesian national antimalarial treatment guidelines were changed in April 2006 from failing regimens including oral quinine, chloroquine and sulphadoxine-pyrimethamine to a universal policy of dihydroartemisinin-piperaquine for uncomplicated malaria due to any *Plasmodium* species and intravenous artesunate for severe malaria. Concomitantly, the recommended total dose of primaquine was increased from 3.5mg/kg to 7mg/kg.

In April 2004, a malariometric surveillance system was created to enable an assessment of the impact of the policy change on various clinical, social and operational outcomes. This system included prospective data collection from community clinics and the local hospital, periodic cross-sectional school and community prevalence surveys and entomological and meteorological data collection. The following outlines the *a priori* plan for the impact assessment.

# Outcomes of interest

## Community

- 1. Change in the absolute number and proportion of blood films read at community clinics within the formal sector that were positive for *Plasmodium* parasitemia, stratified by *Plasmodium* species
  2. Change in the absolute number and proportion of blood films read at community clinics within the formal sector that were positive for *Plasmodium* gametocytes, stratified by *Plasmodium* species
  3. Change in the ratio of *P. vivax* mono- or mixed infection to *P. falciparum* infection for all preceding community outcomes

## Hospital

- 1. Change in the absolute number and proportion of presentations to Mitra Masyarakat Hospital that were associated with microscopically- or RDT-confirmed *Plasmodium* parasitemia, stratified by *Plasmodium* species
  2. Change in the absolute number and proportion of admissions to Mitra Masyarakat Hospital that were associated with microscopically- or RDT-confirmed *Plasmodium* parasitemia, stratified by *Plasmodium* species
  3. Change in the median and aggregated total length of inpatient bed occupancy at Mitra Masyarakat Hospital associated with microscopically- or RDT-confirmed *Plasmodium* parasitemia, stratified by *Plasmodium* species
  4. Change in the absolute number and proportion of deaths at Mitra Masyarakat Hospital that were associated with microscopically- or RDT-confirmed *Plasmodium* parasitemia, stratified by *Plasmodium* species
  5. Change in the absolute number and proportion of malaria-associated presentations to Mitra Masyarakat Hospital that were associated with severe anemia (haemoglobin <7g/dL), stratified by *Plasmodium* species
  6. Change in the ratio of *P. vivax* mono- or mixed infection to *P. falciparum* infection for all preceding hospital outcomes
  7. Proportion of total deaths in the community that occurred at Mitra Masyarakat Hospital before and after policy change

## Combined community and hospital

- 1. Change in the estimated overall incidence of malaria in the region, stratified by *Plasmodium* species
  2. Change in the estimated overall incidence of malaria-related deaths in the region, stratified by *Plasmodium* species
  3. Change in the ratio of *P. vivax* mono- or mixed infection to *P. falciparum* infection for all preceding hospital outcomes
  4. Change in the proportion of patients with malaria estimated to have sought treatment within the formal sector and therefore to have been captured by the surveillance system

# Available Data

## Community

- 1. Entomology – At least weekly human-landing mosquito collections at 5 sentinel sites with subsequent *Anopheles* species identification (April 2004 - December 2009)
  2. Meteorology – Daily total rainfall measurement at one representative site (April 2004 – December 2009)
  3. Community clinics – Weekly reports on number of blood films examined, number of films positive for asexual and sexual *Plasmodium* parasites and number of patients treated for malaria at each of 22 clinics in the formal sector. Data provided were stratified by *Plasmodium* species and aggregated by the age groups <1 year, 1-5 years, 5-10 years and >10 years (April 2004 – December 2009)
  4. Community surveys – Cluster-randomized, cross sectional community surveys of 825 and 800 households done in 2005 and 2013 respectively. Data collected included prevalence of parasitemia as determined by blood film microscopy, prevalence of fever or history of fever, recent deaths of members of the households and detailed information of treatment seeking behaviour
  5. Censuses – Censuses of the population of Mimika District in 2004 and 2013

## Hospital

- 1. Rumah Sakit Mitra Masyarakat (RSMM) – For all patient presentations to hospital the following were collected between April 2004 and December 2013:
     1. Demographic information including sex, age and ethnicity
     2. Hospital department
     3. Date of presentation and discharge
     4. Presence or absence of *Plasmodium* parasitemia as determined by microscopy or RDT, stratified by species
     5. Antimalarial treatment prescribed
     6. Results of full blood count analysis if done
     7. Vital status at the time of discharge
  2. Rumah Sakit Umum Daerah (RSUD) – the following data aggregated by month were provided for patients with malaria confirmed by microscopy or RDT between January 2010 and December 2013:
     1. Sex and age group (<1 year, 1-5 years, 5-10 years, 10-15 years, 15-55, years, >55 years)
     2. *Plasmodium* species
     3. Hospital department
     4. Deaths

# Major potential sources of confounding

1. Changes in the total population and population structure of Mimika District
2. Changes in the proportion of patients seeking treatment at facilities within the surveillance network
3. Opening of RSUD hospital in January 2010 and subsequent provision of both inpatient and outpatient care to patients with malaria
4. Changes in vector control activities and insecticide-treated bednet usage over the study period
5. Changes in mosquito vector abundance
6. Changes in climatic factors, particularly rainfall

# Analysis Plan

## Graphical presentations

For the purposes of graphical presentation of results, prospectively-collected surveillance data will be aggregated by calendar month from April 2004 until the cessation of data collection. The following list of graphs will be produced:

### Community

1. Estimated *Anopheles* mosquito bites per year presented for the three main *Anopheles* mosquito vectors (*An. koliensis*, *An. punctulatus* and *An. farauti* complex) by month
2. Mean daily rainfall by month
3. Absolute number of slides positive for asexual *Plasmodium* parasites at community clinics within the surveillance network by calendar month and *Plasmodium* species with the proportion of cases due to *P. vivax* mono- or mixed infection overlayed
4. Proportion of blood films read at the community clinics within the surveillance network that were positive for *Plasmodium* gametocytes by *Plasmodium* species and month
5. Population structure of the two household surveys with stratification by *Plasmodium* species

### Hospital

Graphs of hospital data will be restricted to data from RSMM as RSUD only opened in January 2010 and data collection procedures did not match those at RSMM and therefore did not allow pooling of data.

1. Absolute number of oral blood schizontocidal prescriptions by drug and month
2. Absolute number of primaquine prescriptions by dose (none, single dose, low dose (total dose ≥1.5mg/kg to <5mg/kg) or high dose (total dose ≥5mg/kg) and month
3. Absolute number of intravenous blood schizontocidal prescriptions by drug and month
4. Absolute number of outpatient malaria presentations by *Plasmodium* species and month with proportion of cases due to *P. vivax* mono- or mixed infection overlayed
5. Absolute number of malaria-related inpatient admissions by *Plasmodium* species and month with proportion of cases due to *P. vivax* mono- or mixed infection overlayed
6. Proportion of all malaria cases due to the different *Plasmodium* species by month with proportion of cases due to *P. vivax* mono- or mixed infection overlayed
7. Proportion of patients presenting to the hospital who were admitted by presence or absence of *Plasmodium* parasitemia, *Plasmodium* species and month with proportion of malaria-related admissions due to *P. vivax* mono- or mixed infection overlayed
8. Total number of days of inpatient bed occupancy per month by presence or absence of *Plasmodium* parasitemia and *Plasmodium* species with proportion of malaria-related bed-days due to *P. vivax* mono- or mixed infection overlayed
9. Proportion of patients presenting to the hospital who were severely anemic (haemoglobin <7g/dL) by presence or absence of *Plasmodium* parasitemia, *Plasmodium* species and month with proportion of malaria-related cases of severe anemia due to *P. vivax* mono- or mixed infection overlayed
10. Proportion of patients presenting to the hospital who died in hospital by presence or absence of *Plasmodium* parasitemia, *Plasmodium* species and month with proportion of malaria-related deaths due to *P. vivax* mono- or mixed infection overlayed
11. Proportion of total malaria-related hospital presentations due to *P. vivax* mono- or mixed infection by age group (<1 year, 1-5 years, 5-15 years, >15 years) and month

## Statistical comparisons

For the purposes of statistical comparisons of prospectively-collected surveillance data and the household surveys, the study period will be divided into 4 phases as follows:

1. Phase 1 – Pre-policy change period – April 2004 to March 2006 (24 months)
2. Phase 2 – Early transition period – April 2006 to March 2008 (24 months)
3. Phase 3 – Late transition period – April 2008 to December 2009 (21 months)
4. Phase 4 – Post transition period – January 2010 to December 2013 (48 months)

**Phase 1** represents the entire period of surveillance prior to policy change.

**Phase 2** represents a two-year period during which it is expected that the bulk of treatment-seeking behaviour change will have occurred and thus the impact of the policy change will have been in a state of flux.

**Phase 3** represents a period during which treatment seeking behaviour was likely to have been relatively constant and the effects of the policy change should have been apparent.

**Phase 4** commences at the point that community surveillance data collection ceased and RSUD hospital opened.

In order to avoid the confounding effects of the latter, before-and-after comparisons will be made between phase 1 and phase 3. Cross sectional prevalence data from the household surveys will be compared between the years 2005 and 2013 as this is when the surveys were done.

Comparisons of binary outcomes will be presented as proportion 1 (n/N) versus proportion 2 (n/N; difference in proportions [binomial 95% confidence interval for difference]) or odds ratios with 95% confidence intervals. Comparisons of incidence rates will be presented as incidence rate 1 (per 1,000 person-years) versus incidence rate 2 (per 1,000 person-years; incidence rate ratio [Poisson 95% confidence interval for difference). Comparisons of length of inpatient stay will be presented as median 1 (interquartile range) versus median 2 (interquartile range). P values will only be presented for comparisons of household survey data as numbers in the community and hospital surveillance are so big that all comparisons will likely reach statistical significance which may be misleading for clinically insignificant results.

# Means of controlling for potential confounding factors

Methods for controlling for the effects of the aforementioned potential confounders will be as follows:

1. *Changes in the total population and population structure of Mimika District*

Incidence rate estimations will take account of population growth by assuming linear growth in population (and therefore person-years of observation) between the censuses done in 2004 and 2013

1. *Changes in the proportion of patients seeking treatment at facilities within the surveillance network*

The estimated proportion of patients with malaria seeking treatment at one of the facilities within the surveillance network in the pre-policy change era and the late transition period have been documented previously (Devine et al AJTMH 2018). The incidence of malaria will be calculated assuming that the surveillance network only captures the proportion of cases determined by the cross sectional surveys.

A sensitivity analysis will be done in which the proportion of malaria patients captured by the surveillance network in the late transition period will be assumed to be the same as during the pre-policy change period (ie no change in treatment seeking behaviour)

1. *Opening of RSUD hospital in January 2010 and subsequent provision of both inpatient and outpatient care to patients with malaria*

Before-and-after comparisons will be limited to the pre-policy change and late transition periods – both of which were prior to the opening of RSUD

1. *Changes in vector control activities and insecticide-treated bednet usage over the study period*

We will be unable to control for changes in vector control activities and bednet usage

1. *Changes in mosquito vector abundance*

A graph of estimated *Anopheles* mosquito bites per year by month will be presented and allowing a visual assessment of the likelihood of confounding

1. *Changes in climatic factors, particularly rainfall*

A graph of mean rainfall by month will be presented allowing a visual assessment of the likelihood of confounding
