# Supplementary figures and images for: Malaria morbidity and mortality following introduction of a universal policy of artemisinin-based treatment for malaria in Papua, Indonesia: A longitudinal surveillance study
Source: PLoS Med. 2019 May 29;16(5):e1002815. doi: 10.1371/journal.pmed.1002815 (PMC6541239; doi:10.1371/journal.pmed.1002815)

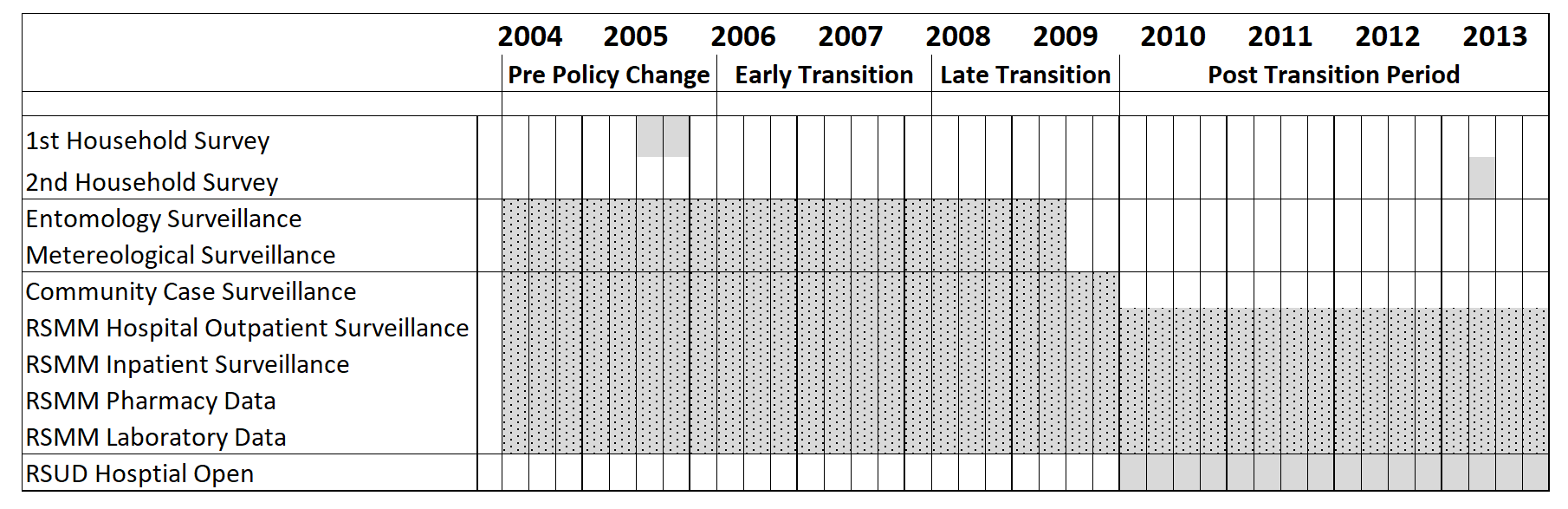

Supplement: S1 Fig — First and second household surveys were reported previously [28]. (TIF) [file pmed.1002815.s003.tif]
