# Supplementary material for: Malaria morbidity and mortality following introduction of a universal policy of artemisinin-based treatment for malaria in Papua, Indonesia: A longitudinal surveillance study
Source: PLoS Med. 2019 May 29;16(5):e1002815. doi: 10.1371/journal.pmed.1002815 (PMC6541239; doi:10.1371/journal.pmed.1002815)
Supplement: S1 Table — (DOCX) [file pmed.1002815.s004.docx]

**S1 Table.**

**List of healthcare facilities within the Mimika District and the periods for which they were within the Malaria Surveillance System (**green = data included in surveillance system, grey = data not included in surveillance system)

|  | **Pre-policy change period** | **Early transition period** | **Late transition period** | **Post transition** |
| --- | --- | --- | --- | --- |
|  | **Apr 2004 - Mar 2006** | **Apr 2006 - Mar 2008** | **Apr 2008 - Dec 2009** | **Jan 2010 - Dec 2013** |
|  |  |  |  |  |
| **Hospitals** |  |  |  |  |
| RSMM |  |  |  |  |
| RSUD |  |  |  |  |
| **Puskesmas clinics** |  |  |  |  |
| Kwampki Lama |  |  |  |  |
| Koprapoka |  |  |  |  |
| Kwamki Baru |  |  |  |  |
| Nawaripi |  |  |  |  |
| Sempan Barak |  |  |  |  |
| SP1 |  |  |  |  |
| SPII |  |  |  |  |
| SPIII |  |  |  |  |
| SPIV |  |  |  |  |
| Timika Indah |  |  |  |  |
| **PHMC clinics** |  |  |  |  |
| Kuala Kencana |  |  |  |  |
| Mile 38 |  |  |  |  |
| Portsite |  |  |  |  |
| Paumako |  |  |  |  |
| Tembagapura |  |  |  |  |
| Hiripau |  |  |  |  |
| Kaugapu Baru |  |  |  |  |
| Mapaurajaya |  |  |  |  |
| Pigapu |  |  |  |  |
| Nayaro |  |  |  |  |
| Paumako |  |  |  |  |
| Asrama & Iwaka |  |  |  |  |
| SP9 |  |  |  |  |
| SPV |  |  |  |  |
| SPVI |  |  |  |  |
| SPVII |  |  |  |  |
| SPXII |  |  |  |  |
| Tipuka |  |  |  |  |
| Utekini Baru |  |  |  |  |
|  |  |  |  |  |
| **Private clinics** |  |  |  |  |
| Klinic Bunda |  |  |  |  |
| Klinic Hadelisari |  |  |  |  |
| Klinic Mandiri |  |  |  |  |
| Klinic Medika |  |  |  |  |
| Klinic Medika Bhakto |  |  |  |  |
| Klinic Restu |  |  |  |  |
| Klinic Teliti |  |  |  |  |
| Klinic Trikora |  |  |  |  |
| Klinic Yahamak |  |  |  |  |
| Praktek Petugas Kehehatan |  |  |  |  |
| Klinic Trakindo |  |  |  |  |
| **Pharmacies** |  |  |  |  |
| Apotik Medika |  |  |  |  |
| Apotik Arguni |  |  |  |  |
| Apotik Etna Farma |  |  |  |  |
| Apotik Kamoro |  |  |  |  |
| Apotik Mega Farma |  |  |  |  |
| Apotik Mimika Farma |  |  |  |  |
| Apotik Mulia |  |  |  |  |
| Apotik Prima |  |  |  |  |
| Apotik Sawito |  |  |  |  |
| Apotik Sinar Intan |  |  |  |  |
| Apotik Yotefa |  |  |  |  |
| Apotik Nindya |  |  |  |  |
